# Pregnancy loss is related to body mass index and prediabetes in early adulthood: Findings from Add Health

**DOI:** 10.1371/journal.pone.0277320

**Published:** 2022-12-01

**Authors:** Yamnia I. Cortés, Shuo Zhang, Jon M. Hussey

**Affiliations:** 1 School of Nursing, University of North Carolina at Chapel Hill, Chapel Hill, North Carolina, United States of America; 2 Carolina Population Center, University of North Carolina at Chapel Hill, Chapel Hill, North Carolina, United States of America; 3 Department of Maternal and Child Health, University of North Carolina at Chapel Hill, Chapel Hill, North Carolina, United States of America; Texas A&M University College Station, UNITED STATES

## Abstract

Pregnancy loss, including miscarriage and stillbirth, affects 15–20% of pregnancies in the United States (US) annually. Accumulating evidence suggests that pregnancy loss is associated with a greater cardiovascular disease (CVD) burden later in life. However, few studies have evaluated the impact of pregnancy loss on CVD risk factors in early adulthood (age <35 years). The aim of this study was to examine associations between pregnancy loss and CVD risk factors (body mass index, blood pressure, hyperlipidemia, diabetes status) in early adulthood. We conducted a cross-sectional analysis using the public-use dataset for Wave IV (2007–2009) of the National Longitudinal Study of Adolescent to Adult Health (Add Health). Our sample consisted of women, ages 24–32 years, with a previous pregnancy who completed biological data collection (n = 2,968). Pregnancy loss was assessed as any history of miscarriage or stillbirth; and quantified as none, one, or recurrent (≥2) pregnancy loss. Associations between pregnancy loss and each CVD risk factor were tested using linear and logistic regression adjusting for sociodemographic factors, parity, health behaviors during pregnancy, and depression. We tested for interactions with race/ethnicity. A total of 670 women reported a pregnancy loss, of which 28% reported recurrent pregnancy loss. A prior pregnancy loss was related to a 3.79 (kg/mm^2^) higher BMI in non-Hispanic Black women, but not white women. Women with recurrent pregnancy loss were more likely to have prediabetes (AOR, 1.93; 95% CI, 1.10–3.37, p<0.05) than women with all live births. Findings suggest that pregnancy loss may be associated with a more adverse CVD risk profile in early adulthood, particularly for women who experience recurrent pregnancy loss. This highlights the need for CVD risk assessment in young women with a prior pregnancy loss. Further research is necessary to identify underlying risk factors of pregnancy loss that may predispose women to CVD.

## Introduction

Cardiovascular disease (CVD) is the leading cause of death for women worldwide [1]. Several female-specific risk factors for CVD have emerged including preterm delivery, intrauterine growth restriction, pregnancy loss, and placental abruption [2–4]. Metabolic and hormonal changes associated with pregnancy such as increased cardiac output, insulin resistance, and hypercoagulability may contribute to the development of CVD [5]. While recent studies suggest that pregnancy outcomes may help identify women at increased risk of later life CVD [6, 7], the long-term effects of pregnancy loss on maternal cardiovascular health have received only modest attention.

Pregnancy loss, including miscarriage and stillbirth, affects approximately 15–20% of pregnancies in the United States (US) annually [8]. In studies examining pregnancy losses and CVD, women with a history of miscarriage or stillbirth had a greater risk of subsequent coronary heart disease compared to women with livebirths [9]. Women with repeated pregnancy loss may be at even greater risk of CVD later in life [10]. However, the exact mechanisms underlying the association between pregnancy loss and CVD are uncertain. Risk factors for miscarriage and stillbirth include obesity, diabetes, and elevated blood pressure [8, 11]. Whether pregnancy loss is related to adverse CVD risk factors in young adults, when early CVD risk interventions may be implemented, has been understudied [11]. Additionally, while Black women in the U.S are more likely to experience a pregnancy loss [12], most studies linking pregnancy loss to CVD include fewer than 10% Black women. Thus, the aim of this study was to examine the association of pregnancy loss (miscarriage, stillbirth) to CVD risk factors in a nationally representative sample of young adults (age 18–35 years). Understanding how pregnancy losses are associated with CVD risk factors, particularly in early adulthood, may elucidate the underlying mechanisms linking pregnancy loss to later life CVD and inform future interventions for CVD prevention in women.

## Methods

### Design and sample

We conducted a cross-sectional analysis using Wave IV public-use data of the National Longitudinal Study of Adolescent to Adult Health (Add Health), an ongoing study developed to assess the behavioral, sociocultural, and environmental factors that influence health in adolescence and beyond. The cohort was constructed during 1994–1995 with a nationally representative sample of over 20,000 adolescents aged 12 to 18 years [13] recruited from 80 schools. The study design stratified by urbanicity, region, school type (public or private), racial/ethnic composition, and school size. The current analytic sample consisted of women with one or more pregnancies who were included in the public-use Wave IV data, when participants were between ages 24 and 32 years. The current analytic sample consisted of women with one or more pregnancies who were included in the public-use Wave IV data. We excluded participants with a multiple pregnancy (e.g., twins, triples), since these are more likely to result in pregnancy complications [14]. Accordingly, the total analytic sample in this study includes 2943 individuals who had complete data on all relevant variables.

### Ethical considerations

The Committee for Protection of Human Subjects at the University of California, Berkeley approved the study protocol.

### Pregnancy loss

Pregnancy loss (miscarriage or stillbirth) was assessed for each pregnancy using an interviewer-administered survey at Wave IV. Participants reported number of pregnancies and whether each of their pregnancies ended in a miscarriage, stillbirth, ectopic pregnancy, or live birth. Since a small sample (n = 30) reported a stillbirth, miscarriage and stillbirth were combined into a pregnancy loss variable and categorized in three groups (0, 1, ≥2). Given the small sample size (n = 30) and different pathophysiology, ectopic pregnancies were not evaluated in this analysis.

### CVD risk factors

CVD risk factors including body mass index (BMI, kg/m^2^), systolic and diastolic blood pressure (mmHg), diabetes status, and hyperlipidemia were retrieved from the Wave IV biological dataset. Trained and certified interviewers assessed the weight and height of participants to determine BMI according to standardized protocols [15]. We used the average of three blood pressure measures collected using the Microlife BP3MC1-PC-IB monitor (MicroLife USA, Dunedin, FL), with participants resting in a seated position for 5 minutes beforehand. Dried blood spot samples were used to assay a lipid panel (total cholesterol, high-density lipoprotein cholesterol, total triglycerides), glucose, and glycosylated hemoglobin (HbA1c). Add Health released Wave IV lipid measurements in deciles. Accordingly, hyperlipidemia was defined as any one of the following: being in the top two deciles of measured triglycerides for females, being in the bottom three deciles of high-density lipoprotein cholesterol, self-report of doctor diagnosed hyperlipidemia, or use of antihyperlipidemic medication. Diabetes/pre-diabetes was defined as any of the following: HbA1c levels of 5.7% or greater, self-reported history of diabetes, or use of any antidiabetic medication.

### Covariates

We included information on socio-demographics (age, race/ethnicity, education, annual income, and health insurance), reproductive history (gravidity, parity, smoking and drinking during pregnancy), current smoking, physical activity, and history of depression. Race/ethnicity was collected from in-home interviews at initial enrollment in 1994–1995. All other data were collected from Wave IV (2007–2009).

### Statistical analysis

Analyses were performed in SAS version 9.4 (SAS Institute, Cary, NC) using sample weights and clusters created by Add Health investigators to obtain nationally representative estimates and account for the complex survey design. Descriptive analyses included calculation of weighted means ± SE or median (interquartile range) for continuous variables and frequencies for categorical variables using. Associations between pregnancy loss and each CVD risk factor were examined in bivariate and multivariable analyses of continuous outcomes (BMI, blood pressure) and binary logistic regression for categorical variables (hyperlipidemia, diabetes/prediabetes). Models adjusted for age, race/ethnicity, education, income, number of pregnancies, smoking and alcohol use during pregnancy, physical activity, and history of depression. We included an interaction term to test whether associations between pregnancy loss and CVD risk factors differed by race/ethnicity. The sample size of 2943 provided 98% power for the fully-adjusted complex multivariable model with 10 covariates (socio-demographics, reproductive history, health behaviors, and depression).

## Results

Approximately 23% of participants (n = 670) reported a pregnancy loss, of which 185 reported experiencing recurrent pregnancy losses (Table 1). Age did not differ between groups, with women being on average aged 29 years. Women experiencing a pregnancy loss did not differ in terms of education, race/ethnicity, or income. However, women a recurrent pregnancy losses had a seemingly greater number of pregnancies (0 = 2 [1, 2]; 1 = 3 [2, 4]; ≥ [2, 6], p<0.01) and a higher BMI (0 = 29.2±0.2; 1 = 30.3±0.5; ≥2 30.5±0.5, p = 0.01) than women without a pregnancy loss. The prevalence of depression also differed by pregnancy loss (0 = 16.7%; 1 = 21.4%; ≥ 2 = 25.4%, p<0.01).

**Table 1:** Characteristics of women with at least one prior pregnancy in Add Health, Wave IV, 2007–2009 (weighted means ± SE, median [IQR] or percentages).

Characteristics	Miscarriage or stillbirth (n = 2943)^a^			*P* ^b^
	0 (n = 2273)	1 (n = 485)	≥ 2 (n = 185)	
Current age,	29.2 ± 0.12	29.0 ± 0.16	29.4 ± 0.17	0.12
Race/ethnicity				0.50
Non-Hispanic white	1564 (68.8)	320 (66.0)	129 (69.7)	
Non-Hispanic Black	631 (27.8)	146 (30.1)	53 (28.6)	
Native American or Alaska Native	18 (0.8)	6 (1.2)	2 (1.1)	
Asian American or Pacific Islander	60 (2.0)	13 (2.7)	1 (0.5)	
Education				0.23
< High school	225 (9.9)	45 (9.3)	21 (11.4)	
High school graduate	432 (19.0)	80 (16.5)	39 (21.1)	
Some college or vocational	1113 (49.0)	258 (53.2)	97 (52.4)	
College graduate or more	503 (22.1)	102 (21.0)	28 (15.1)	
Annual income				0.19
<$25,000	406 (19.1)	89 (19.5)	46 (26.6)	
$25,000-$49,999	619 (29.0)	149 (32.6)	49 (28.3)	
$50,000-$74,999	538 (25.2)	115 (25.2)	44 (25.4)	
$75,000-$99,000	311 (14.6)	56 (12.3)	18 (10.4)	
$100,000	257 (12.1)	48 (10.5)	16 (9.2)	
Any health insurance	1774 (78.1)	370 (76.4)	138 (74.6)	0.43
Reproductive history				
Gravidity	2 [1, 2]	3 [2, 4]	4 [2, 6]	**<0.01**
Parity	2 [1, 3]	1 [1, 3]	2 [1, 3]	**<0.01**
Any smoking during pregnancy	186(15.2)	37(15.9)	17(20.7)	0.40
Any alcohol during pregnancy	45(3.7)	7(3.0)	1(1.2)	0.46
Ever smoke	1485 (65.4)	329 (68.0)	134 (72.8)	0.09
Vigorous physical activity in past 24 hours	883(38.9)	204(42.1)	87(47.0)	0.05
History of depression	379(16.7)	104(21.4)	47(25.4)	**<0.01**
BMI (kg/mm^2^)	29.2±0.2	30.3±0.5	30.5±0.5	**0.01**
Systolic blood pressure (mmHg)	124.6±0.4	124.0±0.7	123.8±0.9	0.58
Diastolic blood pressure (mmHg)	79.2±0.3	79.1±0.6	79.1±0.7	0.97
Highest decile of total cholesterol	207(10.1)	55(12.5)	18(10.7)	0.35
Highest decile of LDL-c	185(9.7)	46(11.2)	18(11.5)	0.53
Hypertension				0.85
Normal	813(36.4)	179(38.1)	66(36.1)	
Prehypertension	1032(46.2)	211(44.9)	85(46.4)	
Hypertension I	304(13.6)	60(12.8)	28(15.3)	
Hypertension II	85(3.8)	20(4.3)	4(2.2)	
Diabetes status				0.06
Normoglycemia	1449(63.7)	301(62.1)	101(54.6)	
Prediabetes	657(28.9)	151(31.1)	62(33.5)	
Diabetes	167(7.3)	33(6.8)	22(11.9)	

*Note*. BMI = body mass index, IQR = interquartile range, LDL-c = low-density lipoprotein cholesterol, SE = standard error. Miscarriage = pregnancy loss at <20 weeks gestation, stillbirth = pregnancy loss at ≥20 weeks gestation.

^a^Due to small sample, a history of stillbirth (n = 30) was combined with miscarriage.

^b^*P* value for overall group differences.

Tables 2 and 3 present findings on the associations between pregnancy loss and individual CVD risk factors. A prior pregnancy loss was related to a higher BMI (ß: 0.97, SE:0.45, p<0.05), but not after adjusting for health behaviors during pregnancy (i.e., smoking, alcohol intake). We found a significant interaction between pregnancy loss and race/ethnicity in association with BMI. Among non-Hispanic Black women, a prior pregnancy loss was related to a higher BMI even after adjusting for all covariates (ß: 3.79, SE:1.05, p<0.05). In fully adjusted models, recurrent pregnancy loss was associated with greater odds of prediabetes (OR [95% CI]: 1.93 [1.10, 3.37]). Recurrent pregnancy loss was also related to greater odds of diabetes in age-adjusted models (OR [95% CI]: 1.99 [1.11, 3.56]). We did not find a significant association between pregnancy loss and hyperlipidemia.

**Table 2:** Associations between pregnancy loss, BMI, and blood pressure.

	Body Mass Index, 𝛽(SE)			Systolic BP, 𝛽(SE)			Diastolic BP, 𝛽(SE)		
	0	1	≥ 2	0	1	≥ 2	0	1	≥ 2
**Model 1**	Ref	1.10 (0.45)*	1.29 (0.66)	Ref	-0.51 (0.80)	-0.95 (1.11)	Ref	-0.06 (0.60)	-0.27 (0.79)
**Model 2**	Ref	0.97 (0.45)*	0.68 (0.64)	Ref	-0.36 (0.80)	-0.83 (1.13)	Ref	0.18 (0.61)	-0.14 (0.82)
**Model 3**	Ref	0.56 (0.73)	0.03 (1.00)	Ref	-0.29 (1.03)	1.45 (1.85)	Ref	-0.52 (0.83)	0.92 (1.42)
**Model 4**	Ref	0.48 (0.72)	-0.06 (1.00)	Ref	-0.31 (1.03)	1.38 (1.86)	Ref	-0.55 (0.83)	0.86 (1.43)
PL*Race/ ethnicity									
** NH white**	Ref	0.28 (0.97)	-0.10 (1.10)	Ref	-0.35 (1.08)	1.40 (2.01)	Ref	-0.50 (0.83)	1.16 (1.58)
** NH Black**	Ref	3.79 (1.05)*	1.17 (1.52)	Ref	3.63 (1.89)	2.96 (3.79)	Ref	2.37 (1.86)	0.55 (2.36)

Note. PL = pregnancy loss,

* p<0.05.

Model 1 adjusted for age.

Model 2: Model 1 + race/ethnicity, education, income.

Model 3: Model 2 + gravidity, smoking during pregnancy, alcohol during pregnancy.

Model 4: Model 3 + physical activity, depression.

Interaction: Model 4 + pregnancy loss * race/ethnicity.

**Table 3:** Associations between pregnancy loss, hyperlipidemia, and diabetes status.

	Hyperlipidemia vs. None (OR, 95% CI)			Prediabetes vs. Normoglycemic (OR, 95% CI)			Diabetes vs. Normoglycemic (OR, 95% CI)		
	0	1	≥ 2	0	1	≥ 2	0	1	≥ 2
**Model 1**	Ref	0.97 (0.65, 1.44)	1.00 (0.52, 1.91)	Ref	1.09 (0.87, 1.37)	1.37 (0.94, 2.00)	Ref	0.88 (0.56, 1.37)	1.99 (1.11, 3.56)*
**Model 2**	Ref	1.01 (0.68, 1.51)	1.07 (0.55, 2.09)	Ref	1.10 (0.87, 1.38)	1.34 (0.90l 2.00)	Ref	0.94 (0.59, 1.50)	1.59 (0.86, 2.93)
**Model 3**	Ref	1.21 (0.59, 2.12)	1.87 (0.57, 6.10)	Ref	1.12 (0.72, 1.75)	1.93 (1.10, 3.39)*	Ref	0.84 (0.36, 2.00)	1.59 (0.59, 4.34)
**Model 4**	Ref	1.11 (0.59, 2.08)	1.77 (0.54, 5.82)	Ref	1.11 (0.72, 1.73)	1.93 (1.10, 3.37)*	Ref	0.83 (0.35, 1.96)	1.57 (0.58, 4.31)

Note. PL = pregnancy loss,

* p<0.05.

Model 1 adjusted for age.

Model 2: Model 1 + race/ethnicity, education, income.

Model 3: Model 2 + gravidity, smoking during pregnancy, alcohol during pregnancy.

Model 4: Model 3 + physical activity, depression.

We found no significant interactions for pregnancy loss * race/ethnicity.

## Discussion

Results of this cross-sectional study indicate that pregnancy loss (miscarriage, stillbirth) is associated with a more adverse CVD risk profile in early adulthood, particularly for women who experience recurrent pregnancy loss. The relation of pregnancy loss to BMI and diabetes status persisted after adjusting for important sociodemographic factors and health behaviors during pregnancy. Our findings are consistent with previous studies linking pregnancy loss to modifiable CVD risk factors [16], which may lead to an increased risk of CVD events in later life [9]. Our analysis adds to the scientific evidence by focusing on CVD risk factors in early adulthood (mean age <30 years) in a nationally representative sample.

Multiple studies have reported an association between pregnancy loss and obesity [14]. Due to the cross-sectional design of this analysis, the directionality of the relationship between pregnancy and BMI was not determined. Obesity has been shown to affect the development of the oocyte and embryo implantation [17]. Additionally, obesity is related to other CVD risk factors that increase risk of pregnancy loss such as smoking, hypertension, and diabetes [18]. It is important to note that in the current analysis, the association between pregnancy loss and BMI was more pronounced among non-Hispanic Black women. This finding is particularly important given that Black women in the US are twice as likely to experience a pregnancy loss compared to white women [12]. Recent evidence has highlighted the determinant role of preconception health on pregnancy outcomes as well as long-term risk of chronic diseases. Our findings may further support the importance of assessing CVD risk early in the life course to optimize pregnancy outcomes, and after a pregnancy loss to enhance future cardiovascular health.

We found that women with recurrent pregnancy loss have a nearly two-fold greater odds of prediabetes in early adulthood than those without a pregnancy loss. Our findings agree with previous studies showing that pregnancy loss is predictive of higher rates of type 2 diabetes in later life [19]. Pregnancy is characterized by many physical, hormonal, hematological, and metabolic adaptations to support fetal development. Insulin resistance during pregnancy results in increases in maternal glucose and free fatty acid concentrations, which are necessary for the rapidly growing fetus [20]. Evidence suggests that metabolic disorders, like impaired glucose tolerance and hyperinsulinemia, are associated with a higher risk of pregnancy loss [21]. It is possible that pregnancy loss is a marker of an underlying metabolic disorder, or susceptibility to type 2 diabetes. Women with prediabetes were older, more overweight/obese, and of higher parity, all risk factors for pregnancy loss. While we found a significant association between pregnancy loss and prediabetes, additional follow-up is needed to assess the relationship with type 2 diabetes.

Several limitations should be considered when interpreting our findings. First, because we performed a cross-sectional analysis, causation cannot be determined. Second, limited data on preconception CVD risk factors hampered our ability to fully adjust for shared risk factors. However, we controlled for health behaviors like smoking and alcohol use during pregnancy. These health behaviors as well as a history of depression may be potential mediators in associations between pregnancy loss and CVD risk. Future studies are necessary to examine these mechanisms and provide further recommendations for earlier identification and prevention of CVD risk. Though self-reported pregnancy loss has been found to be reliable in prior studies, misclassification bias cannot be fully ruled out [22]. It is possible some participants had a missed (or silent) miscarriage, which could have underestimated the observed associations. Due to small sample size, we combined history of miscarriage and stillbirth. Moreover, Add Health did not include data on the timing of pregnancy loss. However, we did assess for recurrent pregnancy loss. While this public use dataset did not include Latinos, this study is one of the most racially and ethnically representative analyses associating pregnancy loss to CVD risk.

## Conclusion

Miscarriage and stillbirth are associated with a greater BMI and increased risk of prediabetes in early adulthood. These findings may support either a shared etiology for pregnancy loss and these CVD risk factors, or a direct pathological link between loss and CVD risk. However, further studies are needed to confirm our results and assess underlying mechanisms. Our findings may further support the importance of assessing CVD risk early in the life course to optimize pregnancy outcomes, and after a pregnancy loss to enhance future cardiovascular health.

## References

[1] Benjamin EJ, Muntner P, Alonso A, Bittencourt MS, Callaway CW, Carson AP, et al. Heart disease and stroke statistics—2019 update: A report from the American heart association. Circulation [Internet]. 2019;139(10). Available from: doi: 10.1161/CIR.0000000000000659 30700139

[2] Catov JM, Newman AB, Roberts JM. Association between infant birth weight and maternal cardiovascular risk factors in the health, aging, and body composition study %J Ann Epidemiol. Jan [Internet]. 2007;17:36–43. Available from: doi: 10.1016/j.annepidem.2006.02.007 16843009

[3] Parikh NI, Jeppson RP, Berger JS. Reproductive Risk Factors and Coronary Heart Disease in the Women’s Health Initiative Observational Study %J Circulation. May [Internet]. 2016;133:2149–58. Available from: doi: 10.1161/CIRCULATIONAHA.115.017854 27143682 PMC4889516

[4] Rich-Edwards JW, Fraser A, Lawlor DA, Catov JM. Pregnancy characteristics and women’s future cardiovascular health: an underused opportunity to improve women’s health? Epidemiol Rev [Internet]. 2014;36(1):57–70. Available from: doi: 10.1093/epirev/mxt006 24025350 PMC3873841

[5] Berends AL, de Groot CJ, Sijbrands EJ, Sie MP, Benneheij SH, Pal R, et al. Shared constitutional risks for maternal vascular-related pregnancy complications and future cardiovascular disease. J Hypertension. 2008;51(4):1034–41. doi: 10.1161/HYPERTENSIONAHA.107.101873 18259037

[6] Roberts JM, Catov JM. Pregnancy is a screening test for later life cardiovascular disease: now what? Research recommendations. J Womens Health Issues. 2012;22(2):e123–8. doi: 10.1016/j.whi.2012.01.001 22385899

[7] Cortés YI, Catov JM, Brooks M, El Khoudary SR, Thurston RC, Matthews KA, et al. Pregnancy-related events associated with subclinical cardiovascular disease burden in late midlife: SWAN. Atherosclerosis. 2019;289:27–35 doi: 10.1016/j.atherosclerosis.2019.07.012 31446211 PMC6952268

[8] Miscarriage [Internet]. Marchofdimes.org. [cited 2022 May 8]. https://www.marchofdimes.org/complications/miscarriage.aspx.

[9] Parker DR, Lu B, Sands-Lincoln M. Risk of cardiovascular disease among postmenopausal women with prior pregnancy loss: the women’s health initiative %J Ann Fam Med. Jul [Internet]. 2014;12:302–9. Available from: doi: 10.1370/afm.1668 25024237 PMC4096466

[10] Kessous R, Shoham-Vardi I, Pariente G, Sergienko R, Holcberg G, Sheiner E. Recurrent pregnancy loss: a risk factor for long-term maternal atherosclerotic morbidity? Am J Obstet Gynecol [Internet]. 2014;211(4):414.e1–11. Available from: doi: 10.1016/j.ajog.2014.05.050 24905415

[11] Magnus MC, Ferreira DDS, Borges MC, Tilling K, Lawlor DA, Fraser A. Cardiometabolic health during early adulthood and risk of miscarriage: a prospective study. Wellcome Open Res. 2020;5:205. doi: 10.12688/wellcomeopenres.16245.2 33644403 PMC7888356

[12] Pruitt SM, Hoyert DL, Anderson KN, Martin J, Waddell L, Duke C, et al. Racial and ethnic disparities in fetal deaths—United States, 2015–2017. MMWR Morb Mortal Wkly Rep [Internet]. 2020;69(37):1277–82. Available from: doi: 10.15585/mmwr.mm6937a1 32941410 PMC7498170

[13] Harris KM, Halpern CT, Haberstick BC, Smolen A. The National Longitudinal Study of Adolescent Health (Add Health) sibling pairs data. Twin Res Hum Genet [Internet]. 2013;16(1):391–8. Available from: doi: 10.1017/thg.2012.137 23231780 PMC3574787

[14] Boiko VI, Nikitina IM, Babar TV, Boiko AV. The problem of miscarriage in multiple pregnancy. Wiad Lek. 2018;71(7):1195–9. 30448784

[15] Hussey JM, Nguyen QC, Whitsel EA, Richardson LJ, Halpern CT, Gordon-Larsen P, et al. The reliability of in-home measures of height and weight in large cohort studies: Evidence from Add Health: Evidence from Add Health. Demogr Res [Internet]. 2015;32:1081–98. Available from: doi: 10.4054/DemRes.2015.32.39 26146486 PMC4487879

[16] Hall PS, Nah G, Vittinghoff E, Parker DR, Manson JE, Howard BV, et al. Relation of pregnancy loss to risk of cardiovascular disease in parous postmenopausal women (from the Women’s Health Initiative). Am J Cardiol [Internet]. 2019;123(10):1620–5. Available from: doi: 10.1016/j.amjcard.2019.02.012 30871746 PMC6488415

[17] Lashen H, Fear K, Sturdee DW. Obesity is associated with increased risk of first trimester and recurrent miscarriage: matched case-control study. Hum Reprod [Internet]. 2004;19(7):1644–6. Available from: doi: 10.1093/humrep/deh277 15142995

[18] Dağ ZÖ, Dilbaz B. Impact of obesity on infertility in women. J Turk Ger Gynecol Assoc [Internet]. 2015;16(2):111–7. Available from: doi: 10.5152/jtgga.2015.15232 26097395 PMC4456969

[19] Virani SS, Alonso A, Aparicio HJ, Benjamin EJ, Bittencourt MS, Callaway CW, et al. Heart disease and stroke statistics-2021 update: A report from the American Heart Association: A report from the American Heart Association. Circulation [Internet]. 2021;143(8):e254–743. Available from: doi: 10.1161/CIR.0000000000000950 33501848 PMC13036842

[20] Egerup P, Mikkelsen AP, Kolte AM, Westergaard D, Rasmussen S, Knop FK, et al. Pregnancy loss is associated with type 2 diabetes: a nationwide case-control study. Diabetologia [Internet]. 2020;63(8):1521–9. Available from: doi: 10.1007/s00125-020-05154-z 32424542

[21] Sonagra AD, Biradar SM, K D, Murthy D S J. Normal pregnancy- a state of insulin resistance. J Clin Diagn Res [Internet]. 2014;8(11):CC01–3. Available from: doi: 10.7860/JCDR/2014/10068.5081 25584208 PMC4290225

[22] Cundy T, Gamble G, Neale L, Elder R, McPherson P, Henley P, et al. Differing causes of pregnancy loss in type 1 and type 2 diabetes. Diabetes Care [Internet]. 2007;30(10):2603–7. Available from: doi: 10.2337/dc07-0555 17586739

